# Occupational characteristics and risk factors associated with endometriosis among Korean female workers

**DOI:** 10.1371/journal.pone.0292362

**Published:** 2023-10-05

**Authors:** Seunghyun Lee, Seung-Yeon Lee, Wanhyung Lee

**Affiliations:** 1 Department of Occupational and Environmental Medicine, College of Medicine, Gachon University, Incheon, Republic of Korea; 2 Department of Family Medicine, International Healthcare Center, Seoul National University Bundang Hospital, Bundang-gu, Seongnam-si, Gyeonggi-do, Republic of Korea; 3 Department of Preventive Medicine, College of Medicine, Chung-Ang University, Seoul, Republic of Korea; IRCCS Burlo Garofolo Trieste, ITALY

## Abstract

Endometriosis is a chronic and debilitating condition that affects daily working life. Characterization of the factors associated with endometriosis in the working population can facilitate the development of prevention and intervention strategies for those at risk of endometriosis. This population-based retrospective study was conducted using the 2007–2015 National Health Insurance Service–Female Employees database. Overall, 151,386 female workers aged 15–64 years were included in the study. Participants with endometriosis were identified using the diagnosis codes in the claims data. Multivariable Cox regression analyses were used to evaluate the effect of sociodemographic, lifestyle, health, and occupational factors on endometriosis risk. Of the 151,386 participants, 4,457 were diagnosed with endometriosis. The risk of endometriosis was significantly higher in 41–60 years group (HR = 1.47 (95% CI, 1.06–2.04)) and in those with body mass index (BMI) < 18.5 kg/m2 (HR = 1.16 (95% CI, 1.05–1.27)) than 15–20 years group and those with normal BMI, respectively. According to the international standard industrial classification, occupational groups with financial and insurance activities, public administration and defence, compulsory social security, and manufacturing were at a higher risk of endometriosis. Although there was no significant association between the risk of endometriosis and type of work, the cumulative prevalence of endometriosis from 2007 to 2015 continued to rise in office workers, manual workers, and both types of workers together. The risk of endometriosis was closely linked to the occupational characteristics of female workers. This study provides a foundation for developing occupational safety and health guidelines for female workers.

## Introduction

Endometriosis is a common, benign, estrogen-dependent, chronic inflammatory condition characterized by endometrial tissue growth outside the uterus. The symptoms associated with endometriosis vary, including chronic pelvic pain, menstrual irregularities, dysmenorrhea, dyspareunia, dysuria, dyschezia, and infertility [1]. Consequently, endometriosis can lead to a marked deterioration in the lives of individuals by interfering with daily and social activities, sexual and reproductive health, and physical and emotional well-being [2]. Approximately 10% of reproductive-aged women have endometriosis worldwide. The prevalence of endometriosis varies by race and ethnicity, and Asian women are generally more likely to be affected than Caucasians. According to the most recent data, the estimated prevalence of endometriosis in South Korea is 3.5 per 1,000 women [3].

Despite the effect of endometriosis on health and quality of life, the etiology and pathogenesis of endometriosis are not fully understood. Race, ethnicity, body mass index (BMI), lifestyle behaviors (i.e., diet, exercise, alcohol consumption, cigarette smoking, and sleep habits), and menstrual and reproductive characteristics (i.e., early menarche, short menstrual cycles, long menstruation duration, and low parity) have been proposed to be possible risk factors for endometriosis [4]. Occupational and environmental risk factors of endometriosis have been investigated with some studies suggesting that night shift work and exposure to certain chemical agents are linked to endometriosis. Indeed, a large prospective cohort study reported that night-shift work increased the risk of endometriosis in an exposure duration-dependent manner [5]. As endometriosis is a highly estrogen-dependent disorder, increased estrogen levels caused by decreased melatonin production in circadian disrupted night shift workers may promote endometriosis growth [6]. However, most epidemiological and clinical studies have estimated the risk of endometriosis from work-related factors as a single independent variable [7].

Thus, our primary objective was to characterize the various factors associated with endometriosis and estimate their risks in the Korean working population based on the 2007–2015 National Health Insurance Service–Female Employees (NHIS-FEM) database.

## Materials and methods

### Data and study participants

**Fig 1:**
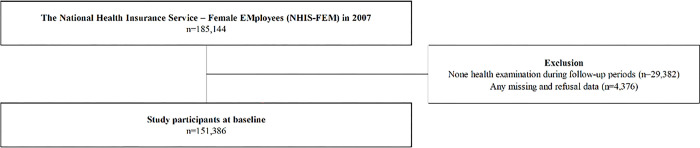
Schematic diagram depicting study participants.

The Korea National Health Insurance Service (NHIS) maintains a database for the entire population of the Republic of Korea with several sub-cohort databases. The NHIS-FEM (2007–2015) was established to understand and evaluate the health status and women-specific disease risk of Korean working women. The NHIS-FEM was started with data on 185,144 working women extracted from approximately 3 million economically active women aged 15–64 years in 2007 [8]. The NHIS-FEM maintains patients’ insurance qualification information and claims for disease diagnosis codes of the Korean Standard Classification of Diseases 7th (KCD-7), correlated with the International Statistical Classification of Diseases and Related Health Problems 10th (ICD-10) by the World Health Organization from 2007 to 2015 [9]. The database had five sections, including general specifications (year, age, gender, region, grade of disability, contribution amount, etc.), health examinations (year, working type, disease history, physical activity, current medications, smoking, drinking, height, weight, blood pressure, laboratory tests, etc.), medical institution (year, location, number of doctors, number of nurses, number of pharmacists, number of beds, etc.), death information (death year and month), and medical examination of cancer (medical examination experience, medical history, year, family history, etc.). Data were anonymized and collected with written informed consent from all the NHIS-FEM participants by the NHIS of Korea. In NHIS-FEM data base, none health examinations during follow-up periods and any missing or refusal data was excluded. The schematic diagram of study participants are shown in Fig 1.

This study was conducted in accordance with the ethical standards of the Declaration of Helsinki (1964) and its subsequent amendments. The data from the NHIS of Korea were collected with written informed consent from all participants. Furthermore, all the data were anonymized to ensure that no individual participants could be identified. This study was approved by the relevant Institutional Review Board (IRB no. GCIRB2020-070).

### Endometriosis

Medical facility visit history from the NHIS-FEM was used to define endometriosis. The endometriosis was defined as patients who had visited a hospital as an inpatient and whose records included KCD-7 diagnosis code ‘N80 Endometriosis’ using diseases claim information based on medical history. This study included participants with the following codes: ‘N80.0 Endometriosis of uterus’; ‘N80.1 Endometriosis of ovary’; ‘N80.2 Endometriosis of fallopian tube’; ‘N80.3 Endometriosis of pelvic peritoneum’; ‘N80.4 Endometriosis of rectovaginal septum and vagina’; ‘N80.5 Endometriosis of intestine’; ‘N80.6 Endometriosis in cutaneous scar’; ‘N80.8 Other endometriosis’; ‘N80.9 Endometriosis, unspecified’.

### Occupational characteristics

We selected three occupational characteristics from the working status and workplace information in the NHIS-FEM. Work type was categorized into two groups: office and manual. In Korea, national health examinations for the working population differ according to the type of work. Office workers are examined biannually, while, manual workers, annually. This can be used to distinguish office workers from manual workers. Work duration was estimated using monthly insurance payment information from the NHIS. We divided working duration into three categories: < 6 months, 7–12 months, and > 12 months. The size of the enterprise based on the number of workers and the International Standard Industrial Classification (ISIC) was collected as the basic information of enterprises in the NHIS-FEM.

### Other variables

Age and household income level were used to demonstrate socioeconomic status based on insurance qualification information. Health status and lifestyle behaviors were included in health examination information. We focused on hypertension, diabetes, anemia, and BMI as health status indicators associated with endometriosis, based on previous studies [10,11]. Lifestyle behaviors included smoking and drinking habits. Previous studies have indicated a close association between endometriosis and lifestyle behaviors [12,13]. Data on smoking and drinking statuses were collected from participants enrolling in national health examination.

### Statistics

Chi-squared test was used to compare the characteristics of participants with and without endometriosis. Hazard ratios (HR) and 95% confidence intervals (CI) for endometriosis were estimated using multivariate Cox regression analyses. For the proportional hazard assumption, a regression model of scaled Schoenfeld residuals against time was assessed for zero slope, and a *p*-value was calculated to be > 0.1, indicating that the proportional hazards assumptions for endometriosis were satisfactory. We estimated the cumulative prevalence of endometriosis according to the type of work during the follow-up year.

The age-standardized prevalence ratio (SPR) and 95% CI of endometriosis according to the ISIC were calculated with all current study participants as the reference group using the indirect standardization method. SPR is the ratio of the observed to expected number of cases. In this study, to estimate the SPR, we stratified age into 5-year groups and calculated the weighted average of age-specific prevalence density rates. Age-specific endometriosis prevalence rates and the number of person-years were estimated for each age group of the entire study population. The expected number of endometriosis cases was calculated after adjusting for age. In the analysis, if SPR and lower limit of 95% CI were > 1, the risk of endometriosis in each ISIC group was considered to be significantly increased. While, for SPR and upper limit of 95% CI < 1, the risk of endometriosis in each ISIC group was considered to be significantly decreased. All analyses were performed using SAS version 9.4 (SAS Institute, Cary, NC, USA).

## Results

Of the 185,144 participants in the NHIS-FEM in 2007, 151,386 participants were selected after excluding 29,382 participants without information about health examinations and 4,376 participants with missing or refusal to share data (Fig 1).

The baseline characteristics of the study participants, with and without endometriosis, are presented in Table 1. Of the 151,386 participants, 4,457 (2.9%) were diagnosed with endometriosis. Endometriosis was most common in the age group of 21–40 years (3.3%), followed by the age group of 41–60 years (2.4%). Participants with endometriosis were found to have a higher income, were more likely to be underweight and anemic, and less likely to have a medical history of hypertension or diabetes. There were no significant differences in smoking or drinking behaviors between the participants with and without endometriosis. In terms of occupational characteristics, endometriosis was more common in office workers and longer-duration workers than in manual workers and shorter-duration workers.

**Table 1:** Baseline characteristics of study participants according to the presence or absence of endometriosis.

	Total participants, *n* (% of column)	Endometriosis, *n* (% of row)		*p*-value
		No	Yes	
Total participants	151,386 (100.0)	146,929 (97.1)	4,457 (2.9)	
**Socioeconomic status**				
Age				<0.0001
15–20	2,385 (1.6)	2,347 (98.4)	38 (1.6)	
21–40	99,173 (65.5)	95,911 (96.7)	3,262 (3.3)	
41–60	48,085 (31.8)	46,932 (97.6)	1,153 (2.4)	
>60	1,743 (1.1)	1,739 (99.8)	4 (0.2)	
Household income level				<0.0001
Low	44,843 (29.6)	43,705 (97.5)	1,138 (2.5)	
Middle–low	38,812 (25.6)	37,807 (97.4)	1,005 (2.6)	
Middle	29,120 (19.2)	28,208 (96.9)	912 (3.1)	
Middle–high	24,226 (16.0)	23,357 (96.4)	869 (3.6)	
High	14,385 (9.5)	13,852 (96.3)	533 (3.7)	
**Occupational characteristics**				
Type of work				<0.0001
Office	78,481 (51.8)	75,959 (96.8)	2,522 (3.2)	
Manual	72,905 (48.2)	70,970 (97.3)	1,935 (2.7)	
Duration of work (month)				0.0060
<6	14,493 (9.6)	14,096 (97.3)	397 (2.7)	
7–12	32,249 (21.3)	31,359 (97.2)	890 (2.8)	
≥12	104,644 (69.1)	101,474 (97.0)	3,170 (3.0)	
Size of enterprise (workers)				0.1712
<5	10,008 (6.6)	9,723 (97.2)	285 (2.8)	
5–29	39,608 (26.2)	38,453 (97.1)	1,155 (2.9)	
30–99	31,488 (20.8)	30,584 (97.1)	904 (2.9)	
100–299	19,389 (12.8)	18,822 (97.1)	567 (2.9)	
≥300	50,893 (33.6)	49,347 (97.0)	1,546 (3.0)	
**Health status**				
Hypertension				<0.0001
No	140,044 (92.5)	135,836 (97.0)	4,208 (3.0)	
Yes	11,342 (7.5)	11,093 (97.8)	249 (2.2)	
Diabetes				0.0293
No	148,361 (98.0)	143,973 (97.0)	4,388 (3.0)	
Yes	3,025 (2.0)	2,956 (97.7)	69 (2.3)	
Anemia				<0.0001
No	117,858 (77.9)	114,850 (97.4)	3,008 (2.6)	
Yes	33,528 (22.1)	32,079 (95.7)	1,449 (4.3)	
Body Mass Index				0.0004
Underweight (<18.5)	15,537 (10.3)	15,028 (96.7)	509 (3.3)	
Normal weight (18.5–24.9)	112,179 (74.1)	108,861 (97.0)	3,318 (3.0)	
Obesity (≥25)	23,670 (15.6)	23,040 (97.3)	630 (2.7)	
**Healthy behavioral**				
Smoking				0.0858
Never or past	147,043 (97.1)	142,695 (97.0)	4,348 (3.0)	
Current	4,343 (2.9)	4,234 (97.5)	109 (2.5)	
Drinking				0.9472
Never or moderate	147,763 (97.6)	143,412 (97.1)	4,351 (2.9)	
Severe	3,623 (2.4)	3,517 (97.1)	106 (2.9)	

The multivariate cox regression results of endometriosis during follow-up period are represented in Table 2. When categorized by socioeconomic status, workers aged 41–60 years had a significantly higher HR, (1.47 (95% CI 1.06–2.04)), than workers aged 15–20 years. Regarding the health status, underweight workers had a higher HR than those with normal BMI (HR = 1.16 (95% CI 1.05, 1.27)).

**Table 2:** Results from the multivariate Cox regression analyses of endometriosis.

	Endometriosis	
	Hazard ratio	95% confidence interval
**Socioeconomic status**		
Age		
15–20	Reference	
21–40	1.06	0.77–1.47
41–60	**1.47**	**1.06–2.04**
>60	1.68	0.60–4.71
Household income level		
Low	Reference	
Middle–low	1.00	0.92–1.09
Middle	1.10	0.99–1.20
Middle–high	1.06	0.96–1.17
High	1.06	0.95–1.19
**Occupational characteristics**		
Type of work		
Office	1.00	0.94–1.07
Manual	Reference	
Duration of work (month)		
<6	Reference	
7–12	0.98	0.87–1.11
≥12	1.02	0.92–1.14
Size of enterprise (workers)		
<5	Reference	
5–29	0.87	0.77–0.99
30–99	0.94	0.82–1.08
100–299	0.90	0.78–1.04
≥300	0.93	0.81–1.06
**Health status**		
Hypertension		
No	Reference	
Yes	0.99	0.87–1.13
Diabetes		
No	Reference	
Yes	1.18	0.93–1.51
Anemia		
No	Reference	
Yes	1.04	0.98–1.11
Body Mass Index		
Underweight (<18.5)	**1.16**	**1.05–1.27**
Normal weight (18.5–24.9)	Reference	
Obesity (≥25)	1.07	0.98–1.17
**Health behavioral**		
Smoking		
Never or past	Reference	
Current	0.98	0.80–1.18
Drinking		
Never or moderate	Reference	
Severe	1.06	0.87–1.28

Note: Statistically significant results are highlighted in bold.

**Fig 2:**
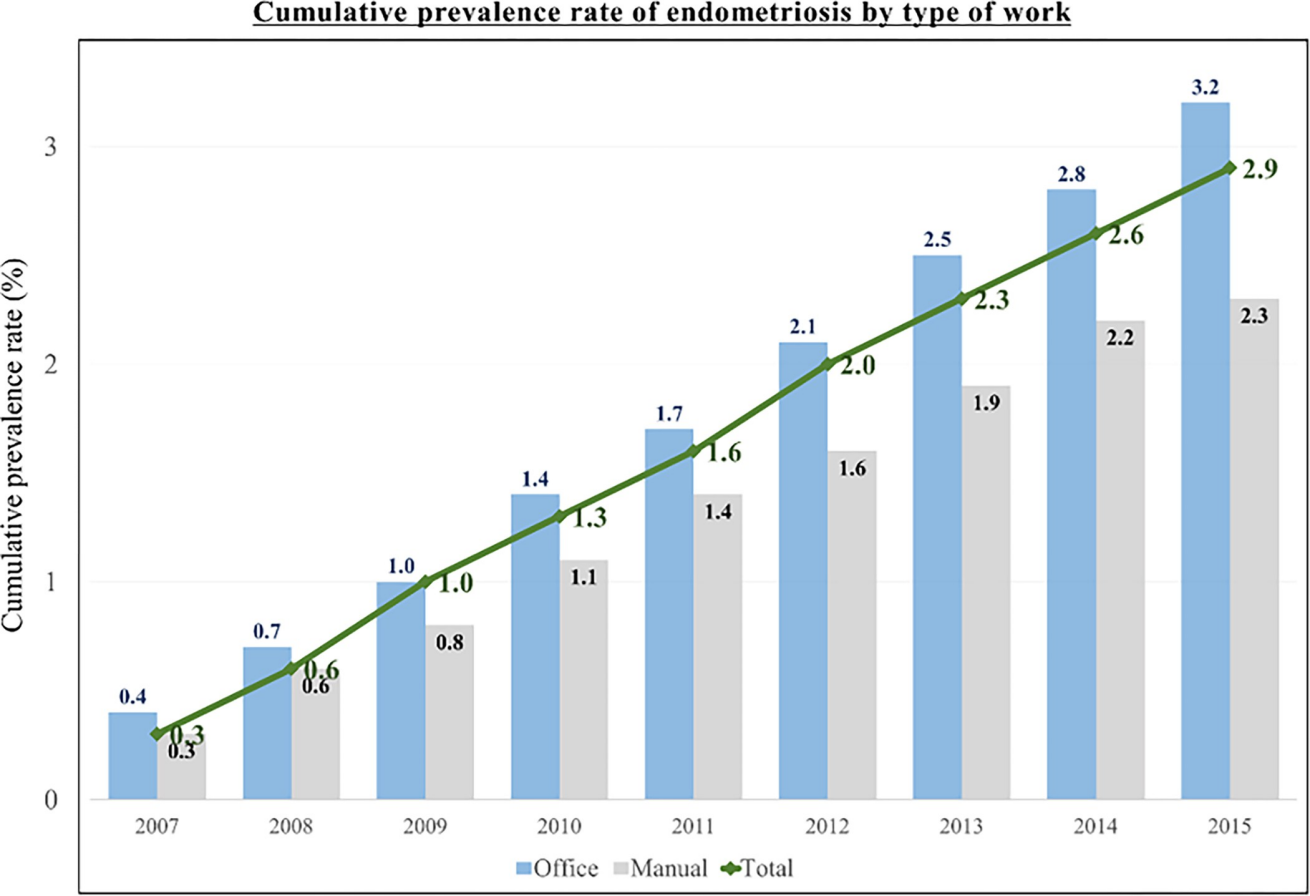
Cumulative prevalence of endometriosis according to type of work during the follow-up period.

There was no significant association between the risk of endometriosis and the type of work. However, the cumulative prevalence rate of endometriosis from 2007 to 2015 continued to rise in office workers, manual workers, and in both types of workers combined, as shown in Fig 2.

**Fig 3:**
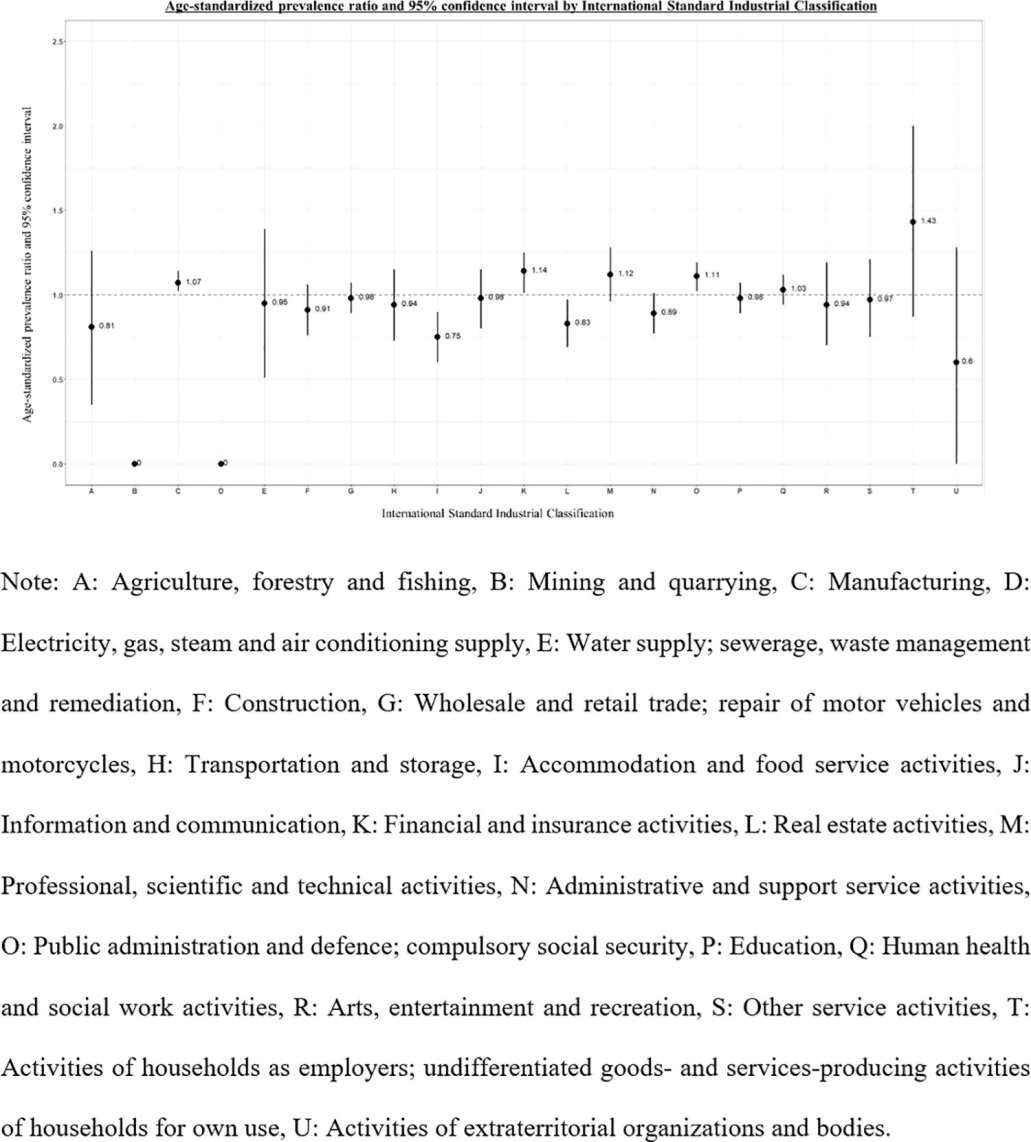
Age-standardized prevalence ratio and 95% confidence interval of endometriosis according to the international standard industrial classification.

Fig 3 illustrates the age-standardized prevalence ratio according to the ISIC. The occupational groups with financial and insurance activities (K), public administration and defence, compulsory social security (O), and manufacturing (C) had significantly higher prevalence ratios.

## Discussion

This study aimed to identify the various factors associated with endometriosis and estimate their risks among the Korean working population using nationwide data obtained from the Korean NHIS.

Endometriosis is a hormone-dependent gynecological disorder occurring commonly during reproductively active age [1]. This was confirmed in this study with a greater prevalence of endometriosis in women aged 21–40 and 41–60 years. Our findings support the notion that endometriosis is reported more frequently in women from higher socioeconomic groups [14]. Women with higher socioeconomic status are more likely to delay childbearing due to a preference for pursuing a career. Delayed childbearing increases the lifetime number of months that women experience menstruation, which may increase the risk of endometriosis, subsequently [4]. Another possible explanation is that inequalities in access to healthcare by socioeconomic groups may cause differences in the possibility of being diagnosed with endometriosis [15].

In the present study, neither cigarette smoking nor alcohol consumption were significantly associated with endometriosis. Smoking has been suggested to have an anti-estrogenic effect [16] which could reduce the risk of endometriosis. However, the association between smoking and the risk of endometriosis remains controversial with some studies reporting an inverse association [17,18], while some report the opposite [12], and others report no significant association [19,20]. In contrast to smoking, alcohol has been suggested to increase circulating bioavailable estrogen levels [21] reducing the risk of endometriosis. However, the association between alcohol use and endometriosis remains under debate. Some studies report that alcohol use is associated with a higher risk of endometriosis [13,22], whereas others have not confirmed this association [20,23]. Since these studies had significant heterogeneity including different study designs, different target populations, and different definitions and measurements of smoking and alcohol consumption, more research is needed to better define the impact of cigarette smoking and alcohol use on the risk of endometriosis.

Although we observed no significant association of hypertension and type 2 diabetes with the risk of endometriosis, some studies have suggested that women with chronic diseases are at a higher risk of developing endometriosis [10]. A recent large-scale prospective cohort study reported that women with hypertension had a higher risk of developing endometriosis than those without hypertension [24]. Inflammation is a major pathophysiological process in various chronic diseases [25]. Additionally, endometriosis is considered a chronic inflammatory condition, with elevated levels of inflammatory markers in the peripheral blood and peritoneal fluid [26]. Inflammation in patients with endometriosis can be triggered by ectopic tissue implants, but it can also promote ectopic proliferation and growth of endometrial tissue [27]. Thus, chronic inflammation associated with chronic diseases can increase the levels of circulating inflammatory markers in the pelvic cavity, increasing the predisposition to endometriosis.

Excessive menstrual bleeding with heavy flow, or intermenstrual bleeding, is one of the most common signs of endometriosis. Other gynecological conditions including uterine fibroids, adenomyosis, and polycystic ovary syndrome, are also associated with excessive menstrual bleeding, and many of them are comorbidities of endometriosis [28,29]. Excessive menstrual bleeding can deplete iron stores, resulting in iron deficiency anemia [11]. In addition to excessive blood loss through menstruation, chronic systemic inflammation is another possible mechanism of anemia in endometriosis [30]. Chronic inflammatory condition is likely to cause iron deficiency anemia.

Low BMI has been reported to be associated with an increased risk of endometriosis [4], with a recent meta-analysis suggesting a 33% reduction in the risk of endometriosis for each 5 kg/m^2^ increase in BMI [31]. We also observed a significantly increased risk of endometriosis in underweight women. The underlying mechanisms of association are unknown; however, several possible explanations have been proposed. Since endometriosis is a chronic, painful condition, many patients use nonsteroidal anti-inflammatory drugs to manage their pain. Chronic use of these medications can cause gastrointestinal side effects, leading to decreased food intake [32]. Painful symptoms and emotional stress associated with endometriosis may also result in loss of appetite and weight loss [33]. Furthermore, obesity is associated with menstrual irregularities and chronic anovulation [34]. This may lower the risk of menstrual regurgitation, of the proposed causes of endometriosis. Another possible explanation for the inverse association between BMI and endometriosis is diagnostic bias. Physicians may be less likely to recommend surgery to obese patients, who are more susceptible to surgical complications. This may reduce the possibility of laparoscopic diagnosis of endometriosis [31].

In terms of work, our study demonstrated that participants with office work, including financial and insurance activities, public administration and defence, and compulsory social security, had a high prevalence of endometriosis. A previous study is consistent with our result suggesting that women’s careers affect the risk of endometriosis [35]. High psychological job demands and decision authority may play a role in stress response. Stress can lead to the release of hypothalamic corticotrophin-releasing hormone, pituitary ACTH, and adrenal cortex glucocorticoids [36]. Thus, perceived work-related stress contributes to changes in hormonal secretions and neuroendocrine disequilibrium, and thus, causing progression of endometriosis.

Moreover, our study demonstrated a significantly high prevalence of endometriosis in “C. Manufacturing” category from the ISIC. Manual workers (i.e., craft workers, plant and machine operators, and assemblers) can be exposed to occupational hazard factors, including harmful chemicals. A recent review suggested a plausible mechanism by which occupational agents may affect endometriosis, involving inflammation and oxidative stress. Occupational chemical exposure induces oxidative stress and a reactive oxygen species imbalance in tissues and cellular components, resulting in damage to membranes, proteins, and DNA [37].

Finally, the “Q. Human health and social work activities” had a significantly high prevalence of endometriosis in the current study. The observed association between occupational history and endometriosis is consistent with the results of a previous study. Marino et al. reported that service station attendants, flight attendants, and health workers, particularly nurses or health aides, had an increased risk of endometriosis [38]. These jobs have round-the-clock service affecting circadian rhythms and reproductive hormones, resulting in endometriosis. Another study assessed the relationship between endometriosis and occupational effects characterizing working status as well as human genotype, but a small size limited the confirmation of the risk of endometriosis by occupational factors [39].

To the best of our knowledge, this study is the first to describe the prevalence of endometriosis and its associated risk factors, including occupational characteristics, among Korean female workers. The study population was derived from a nationwide representative NHIS database, allowing our findings to be generalized to a larger population in South Korea.

Nonetheless, this study may have limitations owing to the nature of the data itself. As the data from the Korean NHIS database were based on information from medical facility visits, it was difficult to include admitted patients. However, the impact of admission data is not significant [40]. Second, the NHIS database was recorded in ICD-10 code format, and our study was limited by the absence of a clinical diagnosis of endometriosis. Further basic and clinical research is needed to overcome these limitations. Finally, our study was limited by the lack of obstetric information (i.e., giving birth, early or late menarche, and contraceptive use), working conditions (i.e., shift work, working hours, and workplace circumstances), exercise level, and dietary factors that might have affected the development of endometriosis. Therefore, further gynecology-based research and epidemiological studies may be required to support these results.

Endometriosis is a gynecological condition that primarily affects women between the ages of puberty and menopause, overlapping the most economically productive years of their lives. Endometriosis has been known to have a substantial negative impact on workplace productivity, with reduced effectiveness at work, additional days of sick leave, and even losing a job. Considering the increasing rate of female participation in the labor force, loss of workplace productivity in women can impose a significant burden on individuals and society. Characterization of the factors associated with endometriosis in the working population can facilitate the development of prevention and intervention strategies for those with or at risk of endometriosis. This study provides a foundation for further research into factors influencing endometriosis in the workplace and informs professionals in occupational medicine or politics on the importance of developing occupational safety and health guidelines for female workers.

## Conclusions

In conclusion, this study found a significant association between endometriosis risk and the occupational characteristics of Korean female workers, providing an overview of general characteristics based on the presence or absence of endometriosis. Furthermore, it offers valuable insights into the age-standardized prevalence rate of endometriosis based on the ISIC through data analysis of the nationwide representative NHIS-FEM database. Our findings will serve as a crucial foundation for future studies, underscoring the need for further research to strengthen the evidence supporting these discoveries.

## Supporting information

S1 Checklist STROBE statement—checklist of items that should be included in reports of observational studies. (DOCX)
